# Influence of centrally mediated symptoms on functional outcomes after carpal tunnel release

**DOI:** 10.1038/s41598-018-29522-3

**Published:** 2018-07-24

**Authors:** Young Hak Roh, Sangwoo Kim, Hyun Sik Gong, Goo Hyun Baek

**Affiliations:** 1 Department of Orthopaedic Surgery, Ewha Womans University Medical Center, Ewha Womans University College of Medicine, 1071 Anyangcheon-ro, Yangcheon-gu, Seoul 07985 South Korea; 2 Department of Orthopaedic Surgery, Seoul National University College of Medicine, Seoul National University Bundang Hospital, 173 Gumi-ro, Bundang-gu, Sungnam 13620 Korea; 3 Department of Orthopaedic Surgery, Seoul National University College of Medicine, Seoul National University Hospital, 101 Daehak-ro, Jongno-gu, Seoul 03080 South Korea

## Abstract

Patients with carpal tunnel syndrome (CTS) often show bilaterally increased pain sensitivity and widespread symptoms. We evaluated the influence of centrally mediated symptoms on functional outcomes of carpal tunnel release (CTR). A total of 120 patients with surgically treated CTS were enrolled. Centrally mediated symptoms were preoperatively measured by administering a self-reported central sensitization inventory (CSI) questionnaire and peripheral sensitization was measured by assessing patient’s pressure pain thresholds (PPT) in the forearm. Boston Carpal Tunnel Questionnaires (BCTQ) were assessed preoperatively and postoperatively at 3 and 12 months. CSI scores slightly correlated with symptom duration and moderately correlated with preoperative BCTQ scores, while PPT slightly correlated with the BCTQ scores. At 3 months, BCTQ symptom and function scores moderately correlated with lower PPTs and higher CSI scores. At 12 months, only severe electrophysiological grade was associated with BCTQ function scores. Multivariable analysis revealed that preoperative PPT, CSI, and female gender were associated with BCTQ scores at 3 months; these factors failed to be associated for 12-month outcomes. Centrally mediated symptoms measured by CSI and peripheral sensitization measured by PPTs correlated with symptom severity and duration. They were associated with poorer functional outcomes after CTR up to 3 months. However, they did not show persistent effects in the long term.

## Introduction

Carpal tunnel syndrome (CTS) is the most common cause of pain due to peripheral nerve entrapment and occurs when the median nerve under the transverse carpal ligament is compressed at the wrist^1^. Reported incidence of CTS ranges from 1 to 5 per 1000 person-years^1^. The number of CTS patients surgically treated in the United States is between 400,000 and 500,000 per year^2^. Although there are many potential causes of increased pressure on the median nerve, idiopathic CTS is the most common one^3^.

Symptoms of CTS are typically restricted to median nerve distribution, yet a significant number of patients with CTS show bilaterally increased pain sensitivity (hyperalgesia) and widespread symptoms^4,5^, demonstrating a generalized disturbance of somatosensory function. CTS might be associated with pain sensitization both at peripheral level and central level^5,6^. Generalized decrease in pressure pain threshold in patients with CTS is associated with pain intensity and duration of symptoms, supporting a role of peripheral drive in initiating and maintaining central sensitization^5^. Functional deficit of nociceptive system has been considered as a pathophysiology of this condition where chronic pain is associated with sensitization in the peripheral afferents, dorsal root ganglion, and the central nervous systems, leading to neuroplastic changes^7^. Studies on subjects with CTS have revealed changes along the afferent pathway in the spinal cord, brain stem, and somatosensory cortex^5,8^.

Research suggests that pain sensitization in peripheral musculoskeletal conditions (such as knee and hip osteoarthritis, shoulder pain, and elbow tendinopathy) is associated with poorer clinical outcomes in response to a surgical or conservative intervention^9–12^. Although the severity of central sensitization in patients with CTS might have contributed to postoperative outcomes of carpal tunnel release (CTR), clinical studies addressing the relationship between preoperative measures of central sensitization and surgical outcomes are lacking. Therefore, the objective of this study was to determine the influence of centrally mediated symptoms on outcomes of CTR.

## Materials and Methods

### Participants

This study was approved by Gil Medical Center Institutional Review Board, and all participants provided written informed consent. This study did not required any deviation of the current clinical practice and was conducted in accordance with the principles of research involving human subjects as expressed in the Declaration of Helsinki (64th, 2013) and with Good Clinical Practice standard. Between January 2014 and October 2017, one surgeon (YHR) surgically treated 143 patients for carpal tunnel syndrome at an urban tertiary referral hospital. Inclusion criteria were: those who were diagnosed with CTS based on clinical symptoms and physical examinations with confirmation by nerve conduction studies, and undertook unilateral CTR. History and symptoms included parasthesia and/or pain in at least two of median nerve-innervated fingers. Other symptoms included weakness and loss of dexterity of the hand. Physical examination for sensory loss, decreased thenar muscle strength, Tinel’s sign, and Phalen’s test were used to reinforce the diagnosis. Electrophysiologic studies were performed prior to surgery. Bland classification^13^ with 7 grades, ranging from grade 0 (normal) to grade 6 (extremely severe) was used in this study based on conduction time and amplitude. Grades 0 and 1 were grouped as mild. Grades 2 and 3 were grouped as moderate. Grades above 3 were grouped as severe. In cases of bilateral involvement, the more severely affected side was chosen for comparative analysis. Exclusion criteria were: those who had severe pain or disability at other joints, previous carpal tunnel surgery, history of psychiatric disorders, peripheral vascular disease, polyneuropathy, cervical radiculopathy, focal nerve entrapment other than carpal tunnel syndrome, pregnancy, hypothyroidism, rheumatoid arthritis, or inability to complete self-reported questionnaire; based on those, 132 (92%) patients were approached for this study. Of those, twelve (9%) were lost to follow up before 12 months, leaving 120 for analysis here (Table 1). Their mean age was 53 years (range, 31–77 years), and 80% (96/120) were women. Thirty-eight (32%) patients had less than high school education. The average duration of symptoms before surgery was 27 months (range, 6–120) (Table 1).

**Table 1:** Demographic and clinical characteristics of participants.

Characteristics	Number or Score
Participants	120
Mean age (years)	53 (31–77)
Male/female	24 (20%)/96 (80%)
Body mass index (kg/m^2^)	27.8 (18.8–34.2)
Less than a high school education	38 (32%)
Affected side (dominant:nondominant)^*^	74:46
Bilaterality (bilateral: unilateral)	36:84
Symptom duration (months)	27 (6–120)
Electrophysiological grade	
Mild	13
Moderate	64
Severe	43
Preoperative functional scores	
BCTQ symptom	2.79 ± 0.65
BCTQ function	2.59 ± 0.58
Pain sensitization measures	
PPT (KPa)	259 ± 40
CSI	34.9 ± 16
Postoperative functional scores	
3 month BCTQ symptom	1.70 ± 0.67
3 month BCTQ function	1.64 ± 0.65
6 month BCTQ symptom	1.41 ± 0.58
6 month BCTQ function	1.32 ± 0.52

Values are expressed with mean ± SD/(range) or number of cases (proportion [%]). ^*^In case of bilateral involvement, the more severely affected side was chosen for comparative analysis.

BCTQ = Boston Carpal Tunnel Questionnaire; PPT = pressure pain thresholds; CSI = Central Sensitization Inventory.

### Pain sensitization measures

Centrally mediated symptoms were preoperatively evaluated using Central Sensitization Inventory (CSI) questionnaire^14,15^. The CSI provides comprehensive information regarding various centrally mediated symptoms^15^, and it demonstrates good reliability^16^ and construct validity^14,15^. This questionnaire assesses 25 somatic and emotional symptoms, which are frequently observed when central sensitization is a contributory factor to chronic pain conditions (e.g. I feel tired and unrefreshed when I wake from sleeping, I am sensitive to bright lights, I have anxiety attacks, Stress makes my physical symptoms get worse, I feel sad or depressed.). The questionnaire also contains pain sensitivity related questions that can be found in everyday life (e.g., My muscles feel stiff and achy, I feel pain all over my body.). Each item was graded with a 5-point scale (0, never; 1, rarely; 2, sometimes; 3, often; and 4, always) representing low to high degree of symptom. The total score ranged from 0 to 100 points.

In addition, peripheral pain sensitization was measured by assessing pressure pain thresholds (PPT)^11,12^. PPT are cut-off points when a sense of pressure changes to pain^17,18^. PPT were assessed in the mid-volar forearm of the affected side using a digital algometer (Somedic, Hörby, Sweden) consisting of a 1-cm^2^ rubber-tipped plunger mounted onto a force transducer. The pressure was applied at a rate of 30 kPa/second. Pressure algometry was repeated three times, and a standardized average of three times of PPT value was used. Although the lack of standardization of the assessment algorithms and the relative paucity of normative data may limit clinical application of PPT^19,20^, the reliability of pressure algometry has been found to be high (intra-class correlation coefficient of 0.91, 95% confidence interval of 0.82–0.97)^21^.

### Procedure

Carpal tunnel release was performed by a hand specialist using the same technique. Local anesthesia with intravenous sedation was used before each operation. After applying tourniquet to the upper arm, a longitudinal incision of about 2–3 cm along the thenar crease was made on the palm 1 cm distal to the wrist crease in line with the 3rd web-space. Transverse carpal ligament was incised and decompressed without neurolysis of the epineurium of the median nerve. The wound was closed with primary cutaneous suture. A short-arm splint was applied for the first 3 days postoperatively. Early active finger motion was performed for all patients.

### Functional outcomes

**Figure 1:**
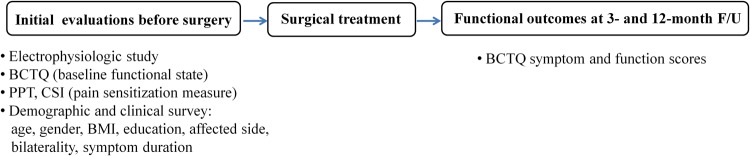
Flow diagram of study protocols. Centrally mediated symptoms were preoperatively measured by administering a self-reported central sensitization inventory (CSI) questionnaire and peripheral sensitization was measured by assessing patient’s pressure pain thresholds (PPT) in the forearm. Boston Carpal Tunnel Questionnaires (BCTQ) were assessed preoperatively and postoperatively at 3 and 12 months. BMI = body mass index, F/U = follow up.

Patients returned for functional assessments at 3 (range, 3–4) and 12 (12–14) months after the surgery (Fig. 1). Assessments of symptoms and functional state of patients were achieved prospectively based on Boston Carpal Tunnel Questionnaire (BCTQ)^22^. The self-administered, patient-based BCTQ has been shown to be reliable, valid, and responsive without notable ceiling of floor effects^23^. It consists of symptom severity scale and functional scale. Symptom severity scale includes 11 items concerning severity, frequency, and duration of symptoms. Functional status scale comprises 8 questions to assess difficulties for eight daily tasks. Each question offers 5 responses in increasing severity. It is scored from 1 (none) to 5 (most severe), and mean values are calculated for all items. Higher scores indicate the presence of more severe symptoms or impairment.

### Statistical analysis

Power analysis indicated that a sample consisting of 120 patients would provide 91% statistical power with α of 0.05 to detect a medium effect size (F^2^ of 0.15) for regression analysis with five main predictors.

Descriptive statistics were used to describe patients’ demographics and clinical characteristics. The confounding variables examined included: age, gender, BMI, education, affected side, bilaterality, symptom duration, electrophysiological grade, and baseline BCTQ scores (Table 2). Kolmogorov-Smirnov test was performed to identify the normality of variable distribution. The relationships between potential predictors and surgical outcomes were determined by using a correlation coefficient for continuous predictor variables (PPT, CSI, age, BMI, education, symptom duration) and using a one-way analysis of variance (electrophysiological grade) or an independent t-test (gender, affected side and bilateral involvement) for categorical potential predictors after normality testing. Bonferroni post hoc methods were used to adjust the p-value from multiple comparisons. Significant predictors with p value < 0.15 in bivariate analysis were selected as candidates for multivariate linear regression analysis to prevent model overfitting. We tested 4 regression models (3 month BCTQ symptoms, 3 months BCTQ function, 12 month BCTQ symptoms, 12 month BCTQ function) each with two steps: the first step included covariates and the second including the sensitization measures. Comparisons of the variabilities accounted for by models (adjusted R^2^) provide measures of the relative influence of explanatory variables on overall variation of the response variable. Categorical variables were dummy-coded with a subgroup for the largest sample size (reference group) Statistical significance was considered when p value was less than 0.05. Statistical analysis was performed using SPSS software version 16.0 (SPSS Inc., Chicago, Illinois).

**Table 2:** Bivariate relationship analysis between potential risk factors and BCTQ symptom and function scores at each measurement time.

Variables	BCTQ at 3months				BCTQ at 12 months			
	Symptom scores	p	Function scores	p	Symptom scores	p	Function scores	p
Gender								
Women (n = 96)	**1**.**8 ± 0**.**6**	**0**.**03**	**1**.**7 ± 0**.**6**	**0**.**04**	1.5 ± 0.6	0.14	1.4 ± 0.6	0.10
Men (n = 24)	**1**.**5 ± 0**.**6**		**1**.**4 ± 0**.**6**		1.3 ± 0.6		1.2 ± 0.5	
Hand dominance								
Dominant (n = 74)	1.7 ± 0.6	0.37	1.6 ± 0.6	0.40	1.6 ± 0.6	0.32	1.4 ± 0.6	0.34
Nondominant (n = 46)	1.6 ± 0.6		1.5 ± 0.6		1.4 ± 0.6		1.3 ± 0.5	
Bilaterality								
Bilateral (n = 36)	1.8 ± 0.6	0.13	1.7 ± 0.6	0.24	1.5 ± 0.6	0.11	1.4 ± 0.6	0.27
Unilateral (n = 84)	1.6 ± 0.6		1.5 ± 0.6		1.3 ± 0.6		1.3 ± 0.6	
Electophysiologic grade^a^								
Mild (n = 13)	1.6 ± 0.6	0.11	1.5 ± 0.5	0.14	1.3 ± 0.5	0.09	1.2 ± 0.5	**0**.**03**
Moderate (n = 64)	1.6 ± 0.6		1.6 ± 0.6		1.4 ± 0.6		1.2 ± 0.6	
Severe (n = 43)	1.8 ± 0.6		1.8 ± 0.6		1.6 ± 0.6		1.5 ± 0.6	
	Correlation coefficient	p	Correlation coefficient	p	Correlation coefficient	p	Correlation coefficient	p
Age	0.12	0.38	0.15	0.42	0.10	0.51	0.03	0.72
BMI	0.25	0.13	0.20	0.30	0.20	0.29	0.18	0.34
Education	0.20	0.32	0.15	0.51	0.12	0.61	0.15	0.55
Symptom duration	0.31	0.12	0.27	0.19	0.34	0.14	0.32	0.17
**PPT**	**0**.**45**	**0**.**02**	**0**.**40**	**0**.**03**	0.35	0.10	0.30	0.13
**CSI**	**0**.**53**	**0**.**01**	**0**.**51**	**0**.**01**	0.37	0.09	0.35	0.10
Baseline BCTQ symptom	0.25	0.22	0.28	0.19	0.27	0.15	0.30	0.11
Baseline BCTQ function	0.32	0.10	0.29	0.13	0.30	0.14	0.37	0.07

PPT = pain pressure thresholds; CSI = Central Sensitization Inventory; BMI = body mass index; BCTQ = Boston Carpal Tunnel Questionnaire.

^a^Bland staging was applied to grade the electrophysiologic severity; grades 0 and 1 were grouped as mild, grades 2 and 3 were grouped as moderate, and grades above 3 were grouped as severe.

### Ethical approval

All procedures performed in studies involving human participants were in accordance with the ethical standards of the institutional and/or national research committee and with the 1964 Helsinki declaration and its later amendments or comparable ethical standards.

## Results

Preoperatively, CSI scores slightly correlated with symptom duration (R = 0.34, p = 0.03) and moderately correlated with BCTQ symptom and function scores (R = 0.45, p = 0.01; R = 0.43, p = 0.01, respectively); PPT slightly correlated with BCTQ symptom and function scores (R = 0.39, p = 0.02; R = 0.36, p = 0.02, respectively).

BCTQ symptom and function scores exhibited significant clinical improvements compared to baseline BCTQ scores (all p < 0.01). The mean BCTQ symptom scores were 1.7 ± 0.7 at 3 months and 1.4 ± 0.6 at 12 months, and the BCTQ function scores were 1.6 ± 0.7 at 3 months and 1.4 ± 0.5 at 12 months, respectively. At 3 months postoperatively, BCTQ symptom and function scores moderately correlated with lower PPT (R = 0.45, p = 0.02; R = 0.40, p = 0.03, respectively) and higher CSI scores (R = 0.53, p = 0.01; R = 0.51, p = 0.01, respectively), and female patients had higher BCTQ symptom and function scores than did males (p = 0.03 and 0.04, respectively) At 12 months postoperatively, only severe electrophysiological grade was significantly associated with BCTQ function scores (p = 0.03) (Table 2).

Multivariable regression analysis revealed that BCTQ symptom and function scores were associated with preoperative PPT (beta = −1.35 [−1.74 to −0.95], p = 0.014; beta = −1.22 [−1.58 to −0.89], p = 0.019, respectively), CSI (beta = 1.61 [0.89 to 2.43], p = 0.008; beta = 1.51 [0.90 to 2.10], p = 0.010, respectively), and female gender (beta = 1.09 [0.77 to 1.38], p = 0.021; beta = 0.95 [0.68 to 1.23], p = 0.025, respectively) at 3 months postoperatively, and these three factors accounted for 35% and 31% of the variance in BCTQ symptom and function scores, respectively. However, BCTQ scores were not associated with any potential predictive factors at 12 months postoperatively (Table 3).

**Table 3:** Stepwise regression analysis for variables predicting BCTQ symptom and function scores after carpal tunnel release.

Model	Variables	Beta	SE Beta	p	R^2^
BCTQ Symptom at 3months	Step 1				
	Gender	1.18		0.029	15%
	Step2				
	Gender	1.09	0.31	0.021	35%
	PPT	−1.35	0.40	0.014	
	CSI	1.61	0.72	0.008	
BCTQ Function at 3months	Step 1				
	Gender	1.02		0.034	13%
	Step 2				
	Gender	0.95	0.28	0.025	31%
	PPT	−1.22	0.36	0.019	
	CSI	1.51	0.60	0.010	
BCTQ Symptom at 12 months	Step 1				
	—				—
	Step 2				
	PPT	0.82	0.39	0.104	—
	CSI	0.95	0.45	0.099	
BCTQ function at 12 months	Step 1				
	EMG grade	1.20	0.57	0.160	—
	Step 2				
	EMG grade	1.31	0.60	0.093	—
	PPT	0.75	0.33	0.119	
	CSI	0.85	0.40	0.135	

PPT = pain pressure thresholds; CSI = Central Sensitization Inventory; BCTQ = Boston Carpal Tunnel Questionnaire; SE = standard error.

## Discussion

Results of our study suggest that pain sensitization measured by CSI and PPT that manifests at different degrees over a continuum correlates with preoperative symptom severity and duration. They were associated with poorer functional outcome scores after carpal tunnel release up to 3 months. However, they did not show persistent effects in the long term (at 12 months).

Our results revealed that preoperative centrally mediated symptoms slightly correlated with BCTQ scores and moderately correlated with CTS symptom duration, consistent with previous findings showing that PPT were negatively correlated with pain intensity and duration of symptoms^24^. Although the median nerve of the affected limb might be more sensitized in individuals with CTS, vibration^25^, thermal^26^, and motor impairments^27^ have been identified bilaterally in patients with unilateral CTS. This suggests that central mechanism might involved in CTS. Increased recruitment of central neurons by peripheral nociceptive inputs, enhanced spatial summation, and increased pain intensity have been suggested to be potential mechanisms involved in pain sensitization^28,29^. Studies on subjects with CTS have revealed changes along the afferent pathway in the spinal cord, brain stem, and somatosensory cortex^8^, decrease in grey matter volume^6^, and loss of spatially segregated representations of digits 2 and digits 3 in the contralateral somatosensory cortex^6,8^.

In this study, centrally mediated symptoms measured by CSI and peripheral sensitization measured by PPT were associated with poor functional outcome at 3 months postoperatively. However it did not show persistent effect at 12 months postoperatively. Although there were no special or intensified treatments after 3 months postoperatively, the influence of pain sensitization on patients’ function and symptom was diminished at 12 months postoperatively. These results are consistent with previous findings showing that physical makeup is important in healthy state, but less important than psychological factors in the context of recovery after upper extremity injuries^30^. This study suggests that physician should attend to patients’ pain sensitization when their impairment or disability is expected to worsen, particularly during early recovery after carpal tunnel release. In this regard, various pharmacological therapies, targeting metabolic factors, cognitive-behavioral therapy, and exercise therapy are effective in improving pain sensitization to reduce symptoms and disability in chronic pain conditions^31,32^. Future research is warranted to determine whether these approaches can enhance treatment outcomes in patients with CTS and severe pain sensitization.

This study has several limitations. First, this study was conducted mostly on female patients with a relatively small sample size. They may not represent the general population with carpal tunnel syndrome. However, our previous epidemiologic study demonstrated that age-adjusted female to male incidence ratio of surgically treated CTS was 5.82 (95% CI, 5.64–6.00)^1^, which was comparable to that in the present study. Second, this study lacked objective measurement for improvement after surgery. Therefore, factors indicating good prognosis might not be based on objective outcome assessment. In addition, large timeframe between the 3- and 12-month postoperative follow-up prohibited the ability to identify when improvement of symptoms and function occurred. Third, identifying pain sensitization clinically is challenging due to the absence of a gold standard. However, quantitative sensory testing is frequently used, and PPT have been shown to be a reliable and sensitive measure of pain sensitization^33^. Fourth, we did not address any psychologic factors such as personal psychologic traits or negative affect that can influence postoperative pain. However, we did exclude patients with a history of psychologic disorders such as depression, psychosis, and somatization disorder from the study. Fifth, 9% of patients were lost during follow-up before the 12-month evaluation, and there were also some missing questions and questionnaires in our cohort. These might have resulted attrition bias. Finally, these patients were limited to a single ethnic population drawn from an urban area of South Korea, and therefore their characteristics and results might not be generalizable to other populations.

## Conclusion

Pain sensitization in patients with CTS measured by PPT and CSI correlated with preoperative CTS symptom severity and duration, and it was associated with poor patient-reported outcomes after CTR up to 3 months postoperatively. Therefore, physicians might be able to improve early recovery after carpal tunnel release by addressing patients’ pain sensitization prior to surgery. However, more research is needed to determine whether early identification and treatment of pain sensitization through pharmacological therapies targeting metabolic factors, cognitive-behavioral therapy, and exercise therapy can enhance treatment outcomes of patients with CTS and severe pain sensitization.
